# Abnormal Spontaneous Discharges of Primary Sensory Neurons and Pain Behavior in a Rat Model of Vascular Dementia

**DOI:** 10.3390/ijms241210198

**Published:** 2023-06-15

**Authors:** Xiaodan Song, Yuchen Wang, Wei Yang, Yingji Wang, Chunjuan Yang, Zhiyong Chen

**Affiliations:** 1 Department of Pharmaceutical Analysis and Analytical Chemistry, College of Pharmacy, Harbin Medical University, Harbin 150081, China; 2 Department of Physiology, School of Basic Medical Sciences, Harbin Medical University, Harbin 150081, China; 3 Department of Inorganic Chemistry and Physics Chemistry, College of Pharmacy, Harbin Medical University, Harbin 150081, China; 4 Department of Anatomy, Histology and Embryology, Institute of Basic Medical Sciences, Neuroscience Center, Chinese Academy of Medical Sciences, School of Basic Medicine, Peking Union Medical College, Beijing 100005, China

**Keywords:** vascular dementia, neuropathic pain, in vivo electrophysiological recording, primary sensory neurons, spontaneous activity

## Abstract

Patients with vascular dementia experience more pain than healthy elders, potentially due to the presence of central neuropathic pain. However, the mechanisms underlying neuropathic pain in vascular dementia remain poorly understood, and there is currently a lack of effective treatment available. In this study, a rat model of vascular dementia was induced by permanently occluding the common carotid arteries bilaterally (2-VO). The cognitive impairments in the 2-VO rats were evaluated using the Morris Water Maze test, while HE and LBF staining were employed to assess brain tissue lesions in the hippocampal, cerebral cortex, and white matter regions known to be associated with severe memory and learning deficits. Furthermore, pain-related behavioral tests, including mechanical and thermal stimuli assessments, were conducted, and in vivo electrophysiological recordings of primary sensory neurons were performed. Compared to sham-operated and pre-operative rats, rats with vascular dementia exhibited mechanical allodynia and thermal hyperalgesia 30 days after surgery. Furthermore, in vivo electrophysiology revealed a significant increase in the occurrence of spontaneous activity of Aβ- and C-fiber sensory neurons in the rat model of vascular dementia. These results indicate that neuropathic pain behaviors developed in the rat model of vascular dementia, and abnormal spontaneous discharges of primary sensory neurons may play a crucial role in the development of pain after vascular dementia.

## 1. Introduction

Vascular dementia (VaD) is a common type of dementia caused by reduced blood flow to the brain, leading to cognitive decline and neurological symptoms [1,2]. Recent studies have indicated that patients with VaD suffer more pain than healthy elderly individuals without cognitive impairment, which might be a reflection of central neuropathic pain (CNP) that markedly impacts their quality of life [3,4,5,6]. CNP, commonly known as post-stroke pain, is a neuropathic pain syndrome [7] characterized by sensory abnormalities and pain in areas corresponding to the injured brain regions caused by cerebrovascular lesions and white matter damage [8]. These white matter lesions also are prominent pathological features of VaD [9,10,11]. Although there have been numerous animal studies focusing on learning deficits, memory, and motor function after VaD, there is limited research on pain associated with VaD. Thus, the mechanisms underlying this symptom remain poorly understood, and effective treatments for pain in VaD have yet to be developed.

Primary sensory neurons of the dorsal root ganglion (DRG), including both A- and C-fiber sensory neurons, transmit pain signals from the peripheral nervous system to the central nervous system. Previous research has demonstrated that, as a pain signal, abnormal discharges of primary sensory neurons play a key role in the development of chronic pain in various conditions [12] and may also contribute to the pathophysiology of CNP in stroke [13,14,15]. However, the roles of these neurons in CNP induced by VaD remain unclear.

The bilateral permanent occlusion of the common carotid arteries (2-VO) model in rats leads to diffuse brain lesions and is characterized by demyelination in the white matter [16] and cognitive impairment [17]. This model is frequently used to simulate the pathological condition of VaD [18,19]. Therefore, we employed the 2-VO rat model to investigate the occurrence of CNP in the VaD model and explore the presence of abnormal discharges in the primary sensory neurons of rats with VaD. Furthermore, we aimed to identify the specific type of neurons associated with pain-related behavior. The findings of our study may contribute to a better understanding of the underlying mechanisms of pain in patients with VaD and potentially guide the development of novel therapeutic approaches for pain management in this population.

## 2. Results

### 2.1. Rat Model of VaD Induced by 2-VO

#### 2.1.1. 2-VO-Induced Cognitive Impairment

**Figure 1:**
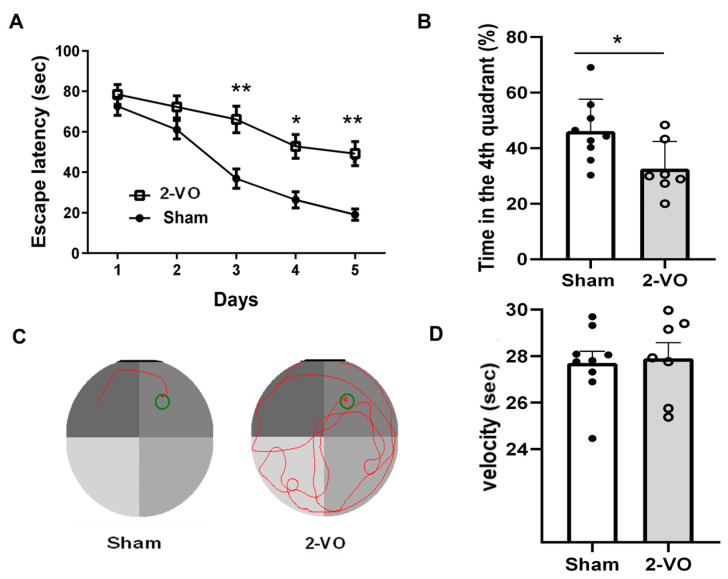
Rats’ performance on the MWM test. (**A**) Escapes latency of rats in successive training trials in sham and 2-VO rats (two-way mixed ANOVA, followed by Sidak’s multiple comparisons test). (**B**) Percentage of time spent in the target quadrant in the probe trial (two-tailed unpaired *t*-test). (**C**) Representative pathways in the last day of training trials. (**D**) Swimming speed in the probe trial (two-tailed unpaired *t*-test). Data are expressed as means ± SEM. * *p* < 0.05; ** *p* < 0.01 vs. Sham.

2-VO-induced cognitive impairments were assessed using the Morris water maze (MWM) test. The escape latencies of both the 2-VO and sham-operated rats were evaluated in a day-dependent manner, and a progressive decline was observed. From day 3, the escape latencies of 2-VO rats were significantly longer compared to the sham-operated rats (Figure 1A, two-way mixed ANOVA, F (1, 8) = 10.52, *p* < 0.05; n = 9 in the sham group and n = 7 in the 2-VO group). During the spatial probe test, the percentage of swimming time in the target quadrant of the 2-VO rats was significantly decreased compared to that of the sham-operated rats (Figure 1B, two-tailed unpaired *t*-test, *p* < 0.05). These findings indicated that 2-VO in rats caused obvious learning and memory impairment. The swimming speed was also evaluated to determine whether the group differences in the escape latency and swimming distance were due to differences in swimming ability. The results showed that there was no significant difference in swimming speed among the experimental groups (Figure 1D, two-tailed unpaired *t*-test, *p* = 0.8145). The representative pathways on the last day of training trials are shown in Figure 1C. These results suggest that the group differences in the escape latency and swimming distance observed in the water maze task were more likely to reflect differences in cognitive and emotional processing rather than differences in physical swimming ability.

#### 2.1.2. 2-VO-Induced Neuropathological Changes in Brain Tissue

2-VO-induced neuropathological changes in brain tissue were assessed using hematoxylin eosin (HE) staining and Luxol fast blue-Cresyl violet (LFB) staining. The histopathological examination was conducted after the MWM test.

**Figure 2:**
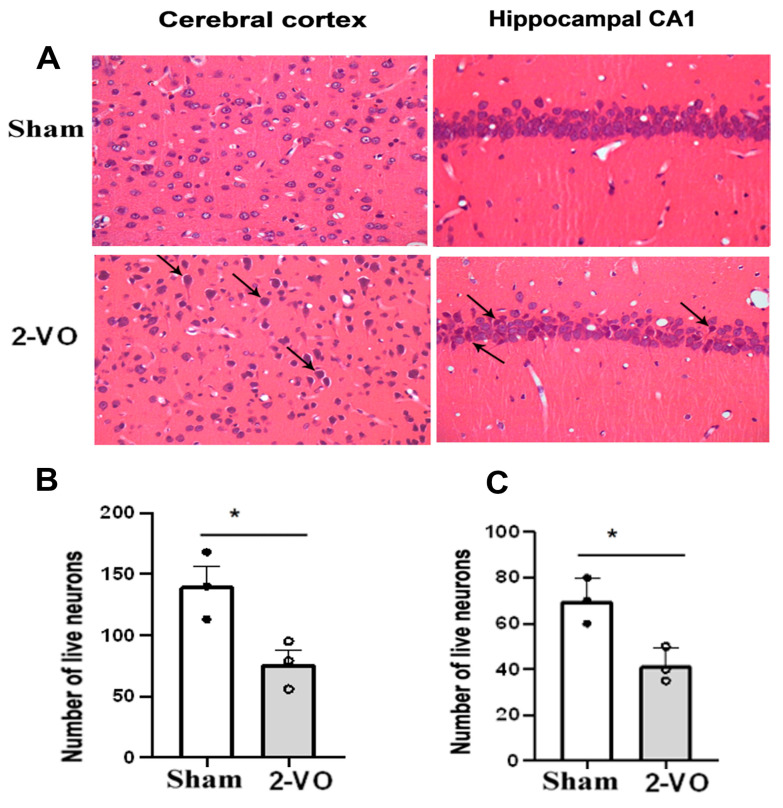
(**A**) Morphological changes in the cerebral cortex and hippocampal CA1 area of rats in each group (HE staining). Quantification analysis of the numbers of live neurons in the cortex (**B**) and the hippocampus (**C**) of rats from each group (two-tailed unpaired *t*-test). Data are expressed as mean ± SEM. n = 3. * *p* < 0.05 vs. Sham. arrow: neuron pyknosis or necrosis (Magnification ×400). The cerebral cortex area is represented by the following stereotaxic coordinates: AP: 2.52 mm, ML: 0.2–0.6 mm, and DV: 2.2–2.4 mm. The hippocampal CA1 area is represented by the following coordinates: AP: −4.36 mm, ML: 1.8–2.2 mm, and DV: 2.2–2.4 mm. These coordinates were obtained from [20].

**Figure 3:**
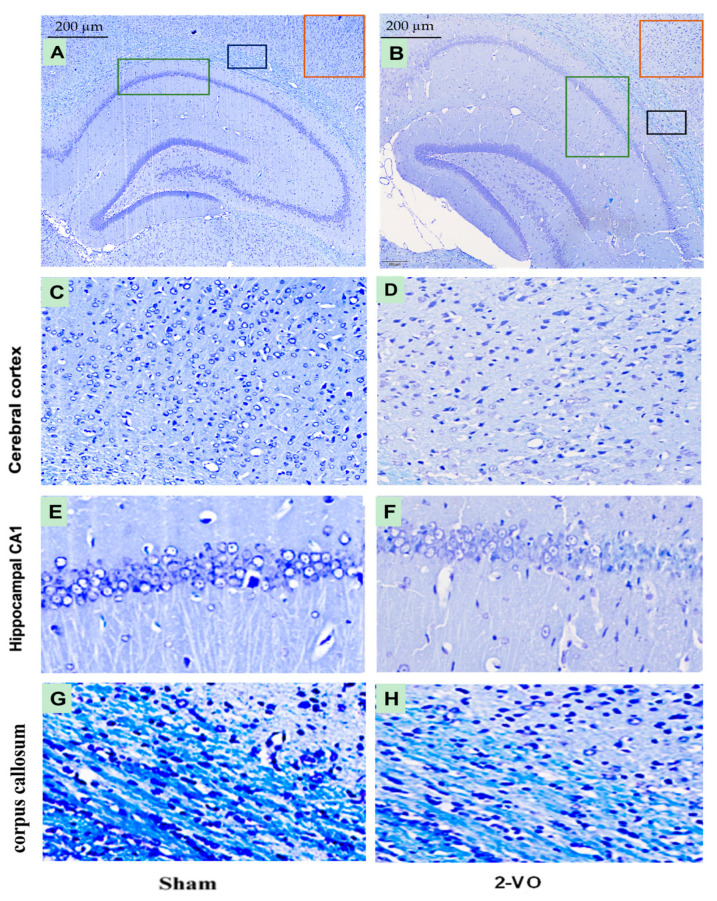
Micrographs showing LFB-stained brain tissue from the Sham and 2-VO groups (**A**,**B**). The red box indicates the magnified part of the cerebral cortex area (**C**,**D**), the green box indicates the magnified part of the hippocampal CA1 area (**E**,**F**), and the black box indicates the magnified part of the corpus callosum area (**G**,**H**). Scale bar: 200 µm (400×). n = 3. The stereotaxic coordinates for the different groups are as follows: Sham group (**C**) AP: −3.84 mm, ML: 4.2–4.6 mm, DV: 2.2–2.4 mm; (**E**) AP: −3.84 mm, ML: 1.8–2.2 mm, DV: 2.2–2.4 mm; (**G**) AP: −3.84 mm, ML: 3.0–3.4 mm, DV: 2.0–2.2 mm; 2-VO group: (**D**) AP: −4.80 mm, ML: 4.8–5.2 mm, DV: 2.6–2.8 mm; (**F**) AP: −4.80 mm, ML: 4.0–4.4 mm, DV: 2.8–3.0 mm; (**H**) AP: −4.80 mm, ML: 4.8–5.2 mm, DV: 3.0–3.2 mm. These coordinates were obtained from [20].

As depicted in Figure 2 and Figure 3, typical neuropathological changes were observed in the cerebral cortex, the CA1 region of the hippocampus, and corpus callosum of rats on day 33 after 2-VO. Notably, the cortical and hippocampal neurons of the rats in the model group exhibited more severe denaturation. The observed denatured neurons exhibited unclear boundaries of the nucleus, karyolysis pyknosis, altered staining properties, swelling or disappearance, vacuolar degeneration of the nucleolus, cytoplasm, and nucleus, as well as dendritic spiral deformation. The density of hippocampal pyramidal cells was reduced, with a looser arrangement and decreased layering. Some cells showed nuclear pyknosis and altered staining, while the gap surrounding the cells became larger. In contrast, the majority of neurons in each brain region of the sham group appeared normal, with clear nucleus boundaries, a uniform distribution of chromatin, distinct nucleoli, and minimal or no gaps around the cells. The hippocampal pyramidal cell layer displayed well-defined and tightly arranged cells with a reduced occurrence of denatured cells (n = 3). Furthermore, the number of live neurons in the cerebral cortex and hippocampal CA1 region was significantly lower in the 2-VO group compared to the sham group (Figure 2B,C, two-tailed unpaired *t*-test, *p* < 0.05).

As shown in Figure 3, the myelin sheath in the corpus callosum of the white matter exhibited a distinct deep blue color, characterized by a densely packed arrangement of myelin fibers in the sham group. However, in comparison to the sham group, the myelin sheath staining in the 2-VO group appeared lighter, indicating a reduction in white matter myelination. Additionally, the myelin sheath structure in the 2-VO group exhibited a looser appearance, suggesting evident demyelination damage in the white matter of the model rats. These findings suggest that 2-VO induces notable neuropathological alterations in brain tissue, which may contribute to the cognitive impairment observed in this model.

### 2.2. Effect of 2-VO on Mechanical and Thermal Hypersensitivity

**Figure 4:**
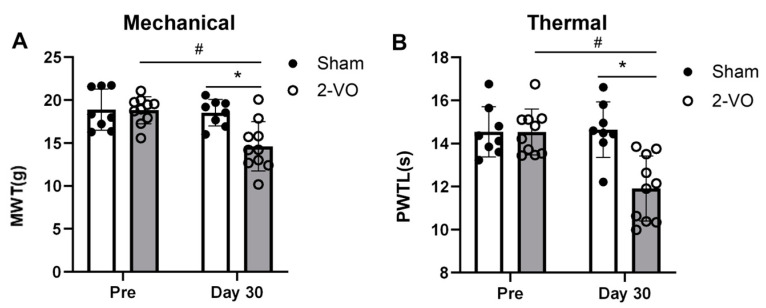
2-VO resulted in mechanical allodynia and thermal hyperalgesia. The nociceptive threshold of rats to mechanical (**A**) and thermal (**B**) stimulation was evaluated on the pre-operative day (pre) and on the 30th day after surgery (day 30) for the 2-VO group (n = 10) and the sham group (n = 8). Two-way mixed ANOVA followed by Tukey multiple comparisons test. Data are expressed as mean ± SEM. * *p* < 0.05, 2-VO rats vs. pre-2-VO rats. ^#^ *p* < 0.05, 2-VO rats vs. sham rats. PWTL: paw withdrawal latency to heat stimulus; MWT: mechanical withdrawal threshold.

As shown in Figure 4, the rats in the 2-VO group (n = 10) exhibited hypersensitivity to both the mechanical withdrawal threshold (MWT) (two-way mixed ANOVA, F_column(1, 9) = 5.791 and F_row(1, 9) = 13.32) and paw withdrawal latency to heat stimulus (PWTL) (two-way mixed ANOVA, F_column(1, 9) = 6.873 and F_row(1, 9) = 17.86) on day 30 after the 2-VO surgery compared to both the sham operation group (n = 8) and the pre-2-VO operation groups (*p* < 0.05). In contrast, there were no significant changes in performance on the MWT and PWTL between the pre- and post-sham operations in the sham group (Figure 4A,B). 

### 2.3. 2-VO Produced Spontaneous Activity (SA) in a Portion of A- and C-Fiber Sensory Neurons In Vivo

**Figure 5:**
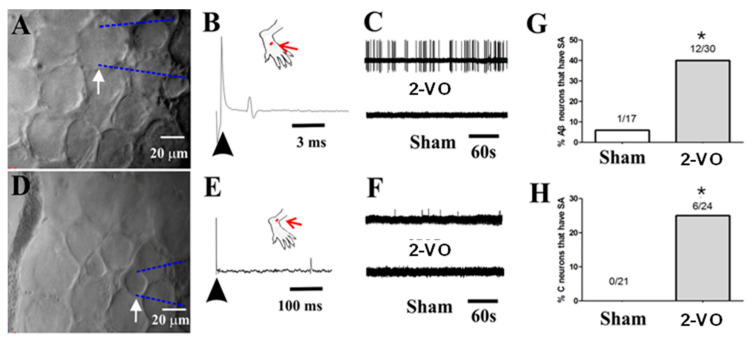
In vivo electrophysiological recordings of DRG neurons. (**A**) Image of a large Aβ-fiber sensory neuron (40 × 40 μm) recording (arrow) with an extracellular electrode. (**B**) An example SA in an Aβ-fiber sensory neuron in a 2-VO rat showing the original extracellular recording trace, and an example of the absence of SA in a neuron from a sham rat is shown below. (**C**) Location of the cutaneous receptive field (red dot) of this neuron on the hairy skin of the hind paw and conduction velocity (upper trace, 26.67 m/s for the Aβ-fiber sensory neuron) obtained using electrical stimulation (arrow) of the receptive field. (**D**) Image of a small C-fiber sensory neuron (20 × 20 μm) recording (arrow) with an extracellular electrode. (**E**) An example SA of a C-fiber sensory neuron in a 2-VO rat with the original extracellular recording trace, and an example the absence of SA in a neuron from a sham rat below. (**F**) Location of the cutaneous receptive field (red dot) of this neuron on the hairy skin of the hind paw and conduction velocity (upper trace, 0.42 m/s for the C-fiber sensory neuron) obtained using electrical stimulation (arrow) of the receptive field. (**G**) Percentages of Aβ-fiber sensory neurons with SA. (**H**) Percentages of C-fiber sensory neurons with SA. * *p* < 0.05, 2-VO rats vs. sham rats. SA: spontaneously active.

After conducting the pain behavior test, in vivo electrophysiological recordings of DRG sensory neurons were performed. The sensory neurons were classified into Aβ-fiber sensory neurons, which had cutaneous receptive fields and fiber conduction velocities ranging from 14.29–33.33 m/s, and responded to mechanical stimuli such as soft brush, hair movement, or gentle pressure. The sensory neurons were also classified as C-fiber sensory neurons, with cutaneous receptive fields and fiber conduction velocities ranging from 0.33–0.89 m/s, which responded to mechanical stimuli such as mild pinching, noxious heat stimulation (51 °C, 5 s), or noxious cold (ice-water, 0 °C, 20 s). The Aβ- and C-fiber sensory neurons were examined by performing extracellular recordings while visualizing them for at least 3 min without any stimuli. The examples of spontaneous activity that were recorded from individual Aβ-and C-fiber sensory neurons are illustrated in Figure 5A–C and Figure 5D–F, respectively. 

A total of 47 Aβ-fiber sensory neurons (17 from sham rats, n = 5 and 30 from 2-VO rats, n = 5) were recorded and analyzed (see Supplementary Table S1). Among the sham rats, only one neuron exhibited SA, resulting in an SA ratio of 5.9% (1/17). In contrast, 12 Aβ-fiber sensory neurons from 2-VO rats exhibited SA, and the SA ratio was 40% (12/30) (Figure 5G). These results showed that the SA ratio of Aβ-fiber sensory neurons in the 2-VO rats was significantly higher compared to the sham rats. A total of 45 C-fiber sensory neurons (21 from sham rats, n = 5 and 24 from 2-VO rats, n = 5) were recorded and analyzed (see Supplementary Table S1). In the 2-VO rats, six C-fiber sensory neurons exhibited SA, and the SA ratio was 25% (6/24) (Figure 5H). No C-fiber neurons exhibited SA in the sham group. These findings indicated that the SA ratio of C-fiber neurons in the 2-VO rats was significantly higher compared to the sham rats. Only one Aδ-fiber sensory neuron (with a conduction velocity of 6.25 m/s) was recorded in the sham group, and none were recorded in the 2-VO group. Therefore, this type of neuron was excluded from the analysis.

## 3. Discussion

Recently, several studies have reported the presence of central neuropathic pain (CNP) in patients with VaD [3,4,5,6,7,21]. However, the specific mechanisms underlying CNP in VaD remain poorly understood. In this study, we used 2-VO rats to establish a VaD model and assessed mechanical allodynia and thermal hyperalgesia on day 30 of 2-VO rats to observe whether VaD induced by 2-VO rats can cause pain behavior. Our findings provide the first evidence that the 2-VO-induced VaD model exhibits mechanical allodynia and thermal hyperalgesia, representing the initial investigation into the development of CNP in this VaD model. Furthermore, our findings demonstrate a significantly higher SA ratio of Aβ- and C-fiber sensory neurons in 2-VO rats compared to sham rats. This suggests that the altered electrophysiological properties of Aβ- and C-fiber sensory neurons in vivo may lead to increased afferent input to the central nervous system [22], thereby playing a crucial role in the development of the hyperalgesia component of CNP in response to mechanical and thermal stimuli. Consequently, our study revealed a novel mechanism of VaD-induced central neuropathic pain, highlighting the potential contribution of increased SA in Aβ- and C-fiber sensory neurons to the development of CNP.

Multiple studies have consistently demonstrated that learning impairments in 2-VO rats progressively worsen over time [23]. Specifically, 2-VO rats exhibit significantly poorer performance in the Morris water maze (MWM) test compared to control rats four weeks after the onset of 2-VO [17]. At this time point, we conducted MWM testing and histological staining using HE and LFB. HE and LFB staining allowed us to evaluate brain tissue lesions in the hippocampus, cerebral cortex, and corpus callosum regions, which are closely correlated with severe memory and learning deficits [17,24]. The results of the study showed that the performances of the 2-VO rats in both the training and probe trials were worse than those of the sham rats, indicating impaired spatial learning and memory four weeks after the 2-VO procedure. Additionally, the histological staining results confirmed the degeneration of neurons in the cerebral cortex, the CA1 region of the hippocampus, and white matter demyelination. These findings provide strong evidence that the 2-VO rats were effective in establishing the VaD model. Therefore, for the subsequent experiments, we utilized the rat model approximately one month after the 2-VO procedure.

Next, we assessed VaD-induced CNP in the 2-VO model. Our results demonstrated a significant decrease in pain thresholds to mechanical and thermal stimuli on day 30 after the 2-VO procedure compared to both the pre- and sham-operated groups, indicating the presence of CNP in the 2-VO rats. To explore the underlying mechanisms of these behavioral changes induced by 2-VO, we examined the electrophysiological properties of primary sensory neurons of the DRG using in vivo electrophysiological recordings in the 2-VO and sham rats [25,26]. Our findings revealed the presence of abnormal discharges of Aβ and C-fiber sensory neurons in the 2-VO rats, which likely contributed to increased central nervous system input and the development of CNP. It is widely known that hypersensitization of primary sensory neurons in the DRG, including A and C-fiber sensory neurons, can cause abnormal sensory phenomena in neuropathic pain, such as dysesthesias/paresthesias [22], tactile allodynia, hyperalgesia, and spontaneous pain [27,28]. Pain signals could activate primary sensory neurons, causing abnormal discharge. Therefore, the observed abnormal discharge in Aβ- and C-fiber sensory neurons under conditions of cerebral ischemic stress may have contributed to the development of mechanical allodynia and thermal hyperalgesia observed in our study.

Although the precise pathophysiological mechanisms underlying the excitability of Aβ- and C-fiber sensory neurons in the 2-VO model are not well understood, it is hypothesized that anatomical and neurochemical changes may contribute to their altered function [8]. Previous studies have shown that the 2-VO model can induce several circulatory components, including inflammatory cytokines such as tumor necrosis factor-alpha and interleukin-1 beta, as well as chemokines [29], which have been reported to be involved in the development of neuropathic pain [30,31]. Therefore, it is possible that the hypersensitization of Aβ- and C-fiber sensory neurons involves the action of inflammatory cytokines or chemokines. Additionally, abnormal changes in ion channels after cerebral ischemia [32,33] and the upregulation of the sodium channel 1.3 subunit or calcium channel α2δ-1 subunit in Aβ- and C-fiber sensory neurons may also be involved in specific hyperexcitability [34,35].

Previously studies have demonstrated that structural and functional changes in certain brain regions can also contribute to the development of hypersensitization in primary afferent neurons, particularly Aβ- and C-fiber sensory neurons, leading to chronic pain [36]. Brain regions such as the white matter, prefrontal cortex, thalamus, hippocampus, insula, and somatosensory cortex have been implicated in the development of hypersensitization, as they are involved in various aspects of pain processing, and they undergo alterations in chronic pain conditions [13,36]. White matter changes in the brain are closely associated with neuropathic pain. In various neuropathic pain conditions, including post-stroke pain and vascular dementia, white matter lesions are considered key factors contributing to the occurrence and exacerbation of pain. These lesions disrupt the normal pathways for transmitting somatosensory information to the brain, resulting in abnormal pain processing and perception. Additionally, white matter changes can disrupt connections between different brain regions, potentially increasing pain sensitivity in patients with vascular dementia [7]. The prefrontal cortex is involved in the cognitive control of pain, including pain modulation and emotional processing. The hippocampus is involved in pain-related memory and learning processes, playing an important role in the development and maintenance of hypersensitivity in chronic pain conditions. Our study also revealed that both white matter lesions and neuronal damage of the cortex and CA1 region of the hippocampus were observed in the brains of the 2-VO model, which is consistent with previous research [37]. These findings suggest that white matter lesions and neuronal damage of the cortex and the hippocampal CA1 region may play a significant role in the hypersensitization of Aβ- and C-fiber sensory neurons and the development of CNP in VaD.

While this study provides insights into the potential development of mechanical hypersensitivity and thermal hyperalgesia in the 2-VO rat model, as well as the potential involvement of abnormal Aβ- and C-fiber sensory neurons firing in the pathogenesis of CNP in VaD, it has some limitations. Firstly, the 2-VO model may not fully replicate the complex pathological changes observed in human VaD, limiting the generalizability of the findings. Secondly, the study did not assess pain behavior longitudinally or investigate the underlying mechanisms of abnormal neuronal firing, calling for further research in these areas. Third, it should be noted that we did not include female rats in our animal model. This decision was made due to several factors, including physiological and behavioral disparities, endocrine influences, and cyclical variations in hormone levels. These dissimilarities and fluctuations had the potential to impact the outcomes of our study, necessitating a comprehensive experimental design and analysis. Given the intricate nature and diverse outcomes of the study, further research is warranted to thoroughly examine the viability of incorporating female rats into the 2-VO animal models. Lastly, the study did not evaluate the effects of potential therapeutic interventions, highlighting the need for future investigations in this domain. While this study provides valuable insights, future research should address these limitations to further enhance our understanding of these findings.

In conclusion, our study provides novel evidence demonstrating the development of mechanical allodynia and thermal hyperalgesia in a rat model of VaD, suggesting the presence of CNP in VaD. We also identified abnormal discharges of Aβ- and C-fiber sensory neurons as a potential mechanism underlying CNP in VaD. Further research studies utilizing this model are warranted to elucidate the detailed mechanisms involved. These findings contribute to our understanding of the pathogenesis of CNP in VaD and have implications for the development of innovative therapeutic drugs and treatment strategies.

## 4. Materials and Methods

### 4.1. Animals

A total of 40 adult male Sprague Dawley (SD) rats were used in the study (200–250 g). The rats underwent the Morris Water Maze (MWM) test, and HE and LFB stains were obtained from the Animal Center of the second Affiliated Hospital of the Harbin Medical University, Harbin, China. The rats used to assess pain behavior and dorsal root ganglion (DRG) sensory neuron recordings were obtained from National Institutes for Food and Drug Control, Beijing, China. All the animals were housed in a room with a controlled environment and a temperature of 23 ± 1 °C, humidity of 55 ± 5%, and a regular 12 h light–dark cycle. The rats were provided with free access to water and food throughout the study. The MWM test, HE, and LFB stains were conducted at the Harbin Medical University and were approved by the ethics committees of the Harbin Medical University. The pain behavioral assessments and DRG sensory neurons recording studies were conducted at the Peking Union Medical College and were approved by the Institutional Animal Care and Use Committees of the Chinese Academy of Medical Sciences and Institute of Basic Medical Sciences (Project #211-2014). All experiments were performed in accordance with the relevant guidelines and regulations. The rats were allowed to acclimatize to the housing conditions for at least four days before the experiments were conducted.

### 4.2. Methods

#### 4.2.1. Animal Model of VaD

**Figure 6:**
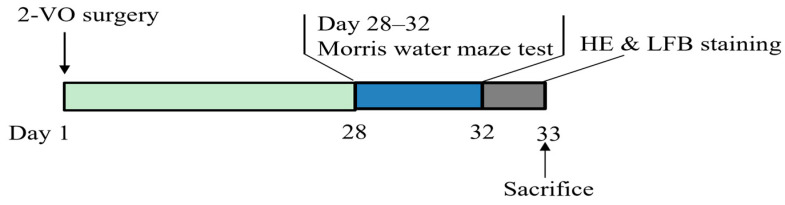
Timeline for surgical procedures (2-VO), Morris water maze (MWM) behavioral test, and Histopathological examination.

VaD was induced in rats using the 2-VO (two-vessel occlusion) method, as described previously [38]. Briefly, male Sprague Dawley rats were anesthetized with 10% chloral hydrate (350 mg/kg). The bilateral common carotid arteries of the rats were separated via a midline cervical incision, and each artery was permanently double ligated with a 5-0 silk suture. The sham-operated animals received the same operation without the ligations. The survival rate using this model establishment method was 85%. The timeline for the surgical procedures (2-VO), Morris water maze (MWM) test, and histopathological examination is shown in Figure 6.

#### 4.2.2. Morris Water Maze (MWM) Test

To validate the 2-VO-induced learning and memory impairment, the spatial learning and memory performance of the rats were assessed using the MWM test, as previous described with minor modifications [39]. The MWM test was performed on day 28 after the surgery. The MWM consisted of a circular tank with a diameter of 2.0 m, filled with opaque water and maintained at a temperature of 23 ± 1 °C. The tank was divided into four equal quadrants. In quadrant IV, a circular platform (10 cm diameter) was submerged 2 cm below the water surface. During the training period, each rat underwent three trials per day for five consecutive days to find the hidden platform. The escape latency, which represents the time taken by the rat to find the platform, was measured and used to assess learning and memory performance. Following the final training trial on day 5, a probe test without the platform was conducted to evaluate spatial memory ability.

#### 4.2.3. Histopathological Examination

Following the MWM test (day 33 after surgery), the rats were deeply anesthetized with chloral hydrate (350 mg/kg, i.p.) and perfused with phosphate-buffered saline (PBS, pH 7.2) followed by 4% paraformaldehyde (Sigma, St. Louis, MO, USA) for fixation. The brains were removed and fixed by immersion in 4% paraformaldehyde for at least 24 h. After fixation, the brains were embedded in paraffin blocks and sectioned serially at a thickness of 5 μm. The neuropathological changes examined in the present study included the cerebral cortex, hippocampal CA1 region, and corpus callosum, and their locations were determined according to the atlas of Paxinos and Watson (2007) [20]. Hematoxylin and Eosin (HE) and Luxol fast blue (LFB) staining were performed on these sections [40]. LFB staining was conducted using a Luxol fast blue–Cresyl violet staining kit (BEIJING SOLARBIO SCIENCE & TECHNOLOGY Co., Ltd., Beijing, China) following the manufacturer’s instructions. Microscopic images of the brain sections were captured using an Olympus BX51 microscope. The numbers of live neurons in the cortex and hippocampus were quantified using a light microscope at 400× magnification using image analysis software (ImageJ, National Institutes of Health, Bethesda, MD, USA, accessed from July 2020 to May 2023, https://imagej.nih.gov/ij/).

#### 4.2.4. Pain Behavioral Testing

To assess pain thresholds, measurements of mechanical and heat stimuli were conducted before and on day 30 after surgery. These tests were performed between 8:00 a.m. and 2:00 p.m. We examined the paw withdrawal response of the rats on their left and right hind paws both before and after surgery. No significant difference was observed between the paws; hence, we computed the mean scores of both hind paws to stimuli.

To evaluate the magnitude of mechanical allodynia, the mechanical withdrawal threshold (MWT) was measured by evaluating the hind paw withdrawal responses. The rats were acclimated for 30 min in acrylic glass boxes with a wire grid bottom. Subsequently, a calibrated Electronic von Frey (Electronic von Frey 2390-5: IITC, Woodland Hills, CA, USA) was applied perpendicularly to the plantar surface of the hind paw, holding it for approximately 3 s. A sharp paw withdrawal was considered as a positive response. We made three measurements per side and calculated the average of these readings as the final data.

Thermal hyperalgesia was measured using the plantar test [41]. A movable radiant heat source located under the glass floor was shone on the hind paw. The maximum automatic cutoff time was set at 20 s to avoid potential tissue damage. The paw withdrawal latency to heat stimulus (PWTL) was measured as the threshold. We averaged the three latency measurements per side as the thermal threshold.

#### 4.2.5. In Vivo Electrophysiological Recording from Primary Sensory Neurons

We performed in vivo extracellular recordings to evaluate the spontaneous activity (SA) of DRG primary sensory neurons, including both A and C-fiber sensory neurons, in a physiologically relevant context. The procedure mirrored our previous methodology, as described in reference [25]. Pentobarbital anesthesia was administered for the operation, starting with an initial dose of 50 mg/kg intraperitoneally, followed by 20 mg/kg/h as required. We removed the L5 transverse process to expose the L4 spinal nerve and then performed a laminectomy at the levels of L1–L6 to expose the L4 DRG. During the surgical process, we periodically dripped oxygenated artificial cerebrospinal fluid (ACSF) onto the ganglia’s surface. The ACSF was comprised of 130 mM NaCl, 3.5 mM KCl, 24 mM NaHCO_3_, 1.25 mM NaH_2_PO_4_, 1.2 mM MgCl_2_, 1.2 mM CaCl_2_, and 10 mM dextrose.

We meticulously removed the sheath covering the surface of the DRG using forceps and scissors under a dissecting microscope. Subsequently, the rat was transferred to a platform attached to the recording table. The spinal process was clamped at the T12 and S1 regions, and a pool was formed by suturing the skin to a metal ring. Then, the ganglion was positioned on a spoon-shaped platform under a microscope (Olympus, BX50WI, Tokyo, Japan). Two platforms, one holding the ganglion and the other holding the rat, were mechanically isolated from each other to allow for manipulation of the lower limb during the search for receptive fields while also ensuring that unwanted mechanical stimuli were not transmitted to the electrophysiologically recorded neuron. The DRG was consistently perfused with oxygenated ACSF at a flow rate of 3–4 mL/min. We maintained the temperature surrounding the ganglion at 35 °C using a heater and a controller (Warner Instrument, TC-324B, Hamden, CT, USA).

To minimize blood flow to the capillary bed in the DRG, a vasoconstricting drug, [Arg8]-vasopressin (10 μM in oxygenated ACSF, Sigma-Aldrich, St. Louis, MO, USA), was applied via a syringe attached to a soft cuff wrapped around the spinal nerve, distal to the DRG, and sealed at each end with Vaseline. We performed extracellular recordings on individual DRG neurons using a micropipette electrode with a tip of 20–25 μm.

Each DRG sensory neuron was classified as a Aβ-, Aδ-, or C-fiber sensory neuron based on its cutaneous receptive field and conduction velocity according to the following method. To identify the cutaneous receptive field of a sensory neuron, the stimuli included innocuous stroking with a cotton-tipped probe, a gentle pinch with the experimenter’s fingers, and von Frey filaments with a fixed tip diameter (200 μm) that were used to deliver different bending forces (for noxious mechanical stimuli). We obtained the conduction velocity by electrically stimulating the cutaneous receptive field with two wire electrodes, or occasionally with a saline-soaked cotton probe. We then calculated the conduction velocity by dividing the latency of the spike peak by the distance between the stimulation electrode and the soma of the recorded neurons. C-fiber sensory neurons had conduction velocities of <2 m/s, whereas Aβ- and Aδ-fiber sensory neurons had conduction velocities of >12 and 2∼12 m/s, respectively [42].

We followed the criteria for classifying a neuron as spontaneously active (SA). For each neuron that was isolated for study, a continuous recording was obtained for a duration of 3 min without applying any external stimulus [42]. If spontaneous ongoing discharge occurred during this period, the neuron was classified as SA. Any injury discharge that occasionally appeared immediately after electrode insertion was ignored.

### 4.3. Statistical Analysis

Data analysis was performed by using Prism 8.0 statistical program (GraphPad Software, Inc., La Jolla, CA, USA). The raw data were first evaluated for Gaussian distribution using the D’Agostino and Pearson test (n > 8) or the KS normality test (n < 8). Normally distributed data were analyzed using parametric statistics. Differences in the escape latency were analyzed statistically using a two-way mixed ANOVA followed by Sidak’s post hoc test. Probe trial performance and the numbers of live neurons in the cortex and hippocampus were analyzed statistically using two-tailed unpaired *t*-tests. The MWT and PWTL data were analyzed statistically using a two-way ANOVA followed by Tukey’s multiple comparisons test. A Chi-square test was used to compare differences in the percentages of neurons with SA. The data were expressed as the mean ± standard error of the mean, or as percentages where appropriate. A *p*-value of *p* < 0.05 was considered significant.

## Data Availability

Not applicable.

## Supplementary Materials

The following supporting information can be downloaded at: https://www.mdpi.com/article/10.3390/ijms241210198/s1.
